# Changes in lower extremity blood flow during advancing phases of pregnancy and the effects of special footwear

**DOI:** 10.1590/1677-5449.002617

**Published:** 2017

**Authors:** Marta Gimunová, Martin Zvonař, Kateřina Kolářová, Zdeněk Janík, Ondřej Mikeska, Radek Musil, Pavel Ventruba, Peter Šagat

**Affiliations:** 1 Masaryk University – FSpS MU, Faculty of Sports Studies, Department of Kinesiology, Brno, Czech Republic.; 2 Masaryk University – LF MU, Faculty of Medicine, Department of Gynecology and Obstetrics, Brno, Czech Republic.; 3 Masaryk University - FN Brno, Faculty Hospital Brno, Department of Gynecology and Obstetrics, Brno, Czech Republic.; 4 Prince Sultan University – PSU, Department of Physical Education, Health and Recreation, Kingdom of Saudi Arabia.

**Keywords:** pregnancy, circulatory system, venous blood flow, popliteal vein, experimental footwear, gravidez, sistema circulatório, fluxo sanguíneo venoso, veia poplítea, calçados experimentais

## Abstract

**Background:**

During pregnancy, a number of changes affecting venous blood flow occur in the circulatory system, such as reduced vein wall tension or increased exposure to collagen fibers. These factors may cause blood stagnation, swelling of the legs, or endothelial damage and consequently lead to development of venous disease.

**Objectives:**

The aim of this study is to evaluate the effect of special footwear designed to improve blood circulation in the feet on venous blood flow changes observed during advancing phases of pregnancy.

**Methods:**

Thirty healthy pregnant women participated in this study at 25, 30, and 35 weeks of gestation. Participants were allocated at random to an experimental group (n = 15) which was provided with the special footwear, or a control group (n = 15). At each data collection session, Doppler measurements of peak systolic blood flow velocity and cross-sectional area of the right popliteal vein were performed using a MySonoU6 ultrasound machine with a linear transducer (Samsung Medison). The differences were compared using Cohen’s d test to calculate effect size.

**Results:**

With advancing phases of pregnancy, peak systolic velocity in the popliteal vein decreased significantly in the control group, whereas it increased significantly in the experimental group. No significant change in cross-sectional area was observed in any of the groups.

**Conclusions:**

Findings in the experimental group demonstrated that wearing the footwear tested may prevent venous blood velocity from reducing during advanced phases of pregnancy. Nevertheless, there is a need for further investigation of the beneficial effect on venous flow of the footwear tested and its application.

## Resumo

**Contexto:**

Durante a gravidez, uma série de mudanças que afetam o fluxo venoso ocorrem no sistema circulatório, tais como menor tensão da parede venosa ou aumento da exposição a fibras de colágeno. Esses fatores podem causar estagnação sanguínea, inchaço das pernas ou dano endotelial e, consequentemente, levar ao desenvolvimento de doença venosa.

**Objetivos:**

O objetivo deste estudo foi avaliar o efeito do uso de calçados especiais projetados para melhorar a circulação sanguínea dos pés sobre as mudanças no fluxo venoso observadas nas fases avançadas da gravidez.

**Métodos:**

Trinta gestantes saudáveis participaram deste estudo às 25, 30 e 35 semanas de gestação. As participantes foram aleatoriamente designadas a um grupo experimental (n = 15) que recebeu calçados especiais, ou um grupo controle (n = 15). A cada sessão de coleta de dados, foram obtidas medidas Doppler do pico de velocidade do fluxo sanguíneo sistólico e da área transversal da veia poplítea direita, utilizando-se um aparelho de ultrassom MySonoU6 com transdutor linear (Samsung Medison). As diferenças foram comparadas utilizando-se o teste d de Cohen para calcular o tamanho do efeito.

**Resultados:**

Nas fases avançadas da gravidez, o pico da velocidade sistólica na veia poplítea diminuiu significativamente no grupo controle, porém aumentou significativamente no grupo experimental. Não houve mudanças significativas na área transversal da veia poplítea em nenhum dos grupos.

**Conclusões:**

Os achados do grupo experimental demonstraram que o uso dos calçados especiais testados pode evitar que a velocidade do fluxo venoso diminua nas fases avançadas da gravidez. No entanto, mais estudos são necessários para investigar os efeitos benéficos sobre o fluxo venoso do uso dos calçados testados e suas aplicações.

## INTRODUCTION

Changes in blood flow during pregnancy are likely to play a role in development of venous insufficiency and thromboembolic events.^1^^,^ 2 Venous insufficiency and varicose disease were observed in 43% and 72.7% of pregnant women, respectively. Additionally, 50% of pregnant women complained of lower limb edema.^3^^,^ 4 One mechanical factor that affects venous return is the growing uterus. In the supine position, the uterus presses on the inferior vena cava, resulting in reduced venous return.^5^^,^ 6

However, the major factors causing pregnancy-related blood vessel changes are pregnancy-related hormonal and physiological changes.^1^ The total volumes of blood, plasma, and erythrocytes increase during pregnancy to provide an increased blood supply to the uterus and placenta. The total blood volume of 4,000 mL prior to pregnancy increases to 5,300 mL at week 36 of gestation. During pregnancy, the number of white blood cells and blood coagulation also increase.^7^ Furthermore, a reduction in vein wall tension can cause stagnation of blood and swelling of the legs and women with a predisposition may develop varicose veins. Additionally, vein dilatation and exposure to collagen fibers can cause endothelial damage and lead to blood clot formation.^1^^,^ 5 ^,^ 8 A previous study shows that blood flow velocity is statistically lower in pregnant women with venous insufficiency. Nevertheless, a substantial reduction in velocity during the last trimester of pregnancy was also observed in healthy pregnant women, reaching its peak at week 36 of gestation.^1^^,^ 2

A number of special types of footwear have been designed to ameliorate pregnancy-related problems, such as foot swelling and increased foot volume or decreased height of foot arches.^9^ Some of the shoes tested are also claimed to have an effect on blood circulation of the feet. For example, balanced inclined shoes were observed to decrease plantar pressure moments and increase bloodstream velocity, suggesting that this type of footwear may both decrease the excessive load on the feet and improve the blood circulation of feet during pregnancy.^10^ The patented footwear and insoles (J Hanák R, Ltd.) used in this study are designed to help redistribute the forces acting on the foot, to support both longitudinal and transverse arches of the foot, and to strengthen the foot muscles during movement and, furthermore, they are claimed to have a positive effect on foot blood supply.^11^ Therefore, the purpose of this study is to evaluate the effects of this special footwear on changes in blood flow velocity and the cross-sectional area of the popliteal vein at 25, 30, and 35 weeks of gestation.

## METHODS

Thirty healthy pregnant women were recruited from the Gynecology and Obstetrics department at the Faculty Hospital of Brno and participated in this study three times during their pregnancies. Participants were allocated at random to an experimental group (n = 15, 30.70 ± 3.82 years of age, mean body height 165.70 ± 6.15 cm, 9 primagravida, others in their second or third pregnancies), which was provided with the special footwear, or a control group (n = 15, 30.94 ± 3.91 years of age, body height 166.57 ± 6.93 cm, 7 primagravida, others in their second or third pregnancies). Mean body mass and the times of the data collection sessions in gestational weeks are shown in Table 1 and 2 for the experimental and control groups, respectively.

**Table 1:** Mean body mass (kg) at the three data collection sessions and their timing (weeks). Experimental group.

**Experimental group**	**Session 1**	**Session 2**	**Session 3**
Gestational week	25.1 (SD = 1.65)	30.3 (SD = 0.85)	35.55 (SD = 0.98)
Body mass	70.20 (SD = 8.62)	74.08 (SD = 8.38)	75.99 (SD = 8.86)

SD: standard deviation.

**Table 2:** Mean body mass (kg) at the three data collection sessions and their timing (weeks). Control group.

**Control group**	**Session 1**	**Session 2**	**Session 3**
Gestational week	24.95 (SD = 1.56)	30.23 (SD = 1.01)	35.30 (SD = 1.03)
Body mass	73.88 (SD = 9.25)	76.99 (SD = 8.82)	79.89 (SD = 9.34)

SD: standard deviation.

Doppler measurements (pulsed-wave) of the right popliteal vein were performed at three pregnancy stages, at 25, 30 and 35 weeks of pregnancy, using a MySonoU6 ultrasound machine with a linear transducer (Samsung Medison®) at the Laboratory of Kinanthropological Research at the Faculty of Sports Studies, Masaryk University, Czech Republic, between February and December of 2016.

Doppler ultrasonography is a simple, noninvasive method, commonly used to study venous hemodynamics.^12^ During Doppler measurements, participants lay on their left sides, with the right leg elevated about 10 degrees and slightly bent at the knee joint. The data measured were peak systolic blood flow velocity (cm/s), when the transducer was parallel with the axis of examined vessel, and the cross-sectional area of the popliteal vein (mm^2^), when the transducer was placed perpendicular to the vein axis. All participants provided written informed consent prior to participation in the study. The study was approved by the Ethics board at the Faculty of Sports Studies, Masaryk University, Brno, Czech Republic.

**Figure 1:**
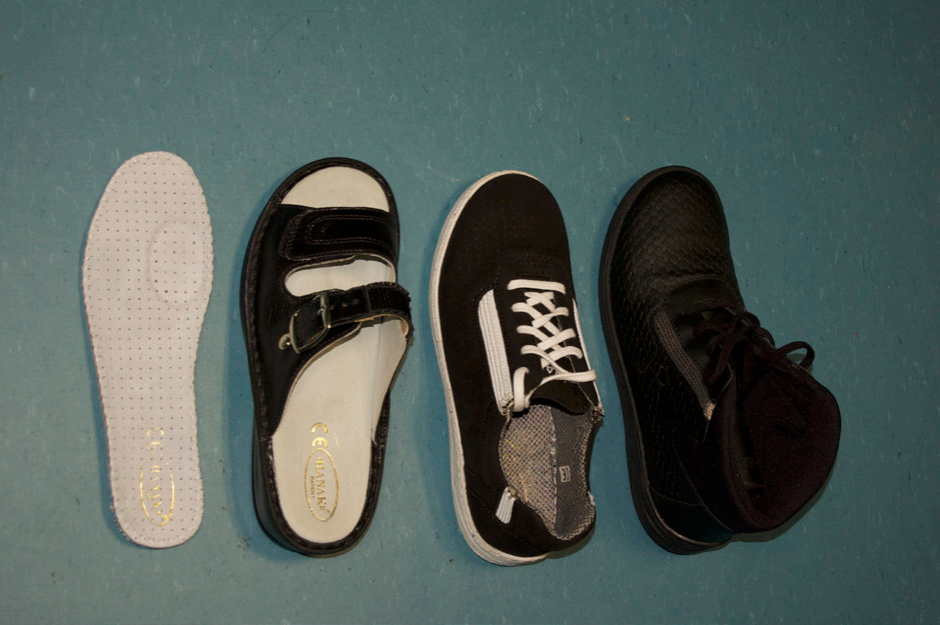
Footwear tested: insoles, slippers, sneakers, and winter shoes.

Participants from the experimental group chose the size and color of two pairs of test footwear - slippers and sneakers or winter shoes (Figure 1), depending on the season - and they were provided with the footwear two weeks after the first measurement and instructed to wear it at least 3 hours per day. At home all participants wore the experimental slippers, while for outdoor walking they wore the sneakers or winter shoes.

The shoes tested contained patented J Hanák R, Ltd. insoles, which are made of pressed cork and their most prominent feature is a depression under the first metatarsophalangeal joint to promote more balanced loading of all toes when walking. This stimulates the muscles and connective tissue structures of the transverse arch. Elastic straps made of leather sewn into the shoe upper sole at the instep and the heel sections provide space for the work of the longitudinal arch of the foot and in the heel region, together with a depression under the heel portion, enabling correction of the position of the calcaneus.^11^^,^ 13

### Statistical analysis

Comparisons were made by effect size because the number of participants is not suitable for statistical analysis. Differences between the experimental and control groups in cross-sectional area and peak systolic velocity (PSV) of the popliteal vein were compared by effect size obtained by calculating Cohen’s d, which interprets the mean differences between measurements taken at the three different stages of pregnancy.

## RESULTS

The mean popliteal vein cross-sectional area and PSV for the experimental and control groups at the three different stages of pregnancy are shown in Table 3. These data show that while there was a reduction in blood flow velocity in the control group, PSV increased in the experimental group during advanced phases of pregnancy. The effect size comparison between the control and experimental group shows that peak systolic velocity in the experimental group was significantly slower at the first measurement, before the special footwear had been worn.

**Table 3:** Mean cross-sectional area (cm^2^) and peak systolic velocity (cm/s ± standard deviation) of popliteal vein in control and experimental groups at three data collection sessions and effect size comparison.

	**Session 1**		**Session 2**		**Session 3**	
	**Area**	**PSV**	**Area**	**PSV**	**Area**	**PSV**
Control group	32.61 ± 12.22	2.80 ± 0.45	32.45 ± 8.11	2.71 ± 0.41	33.76 ± 13.04	2.60 ± 0.28
Experimental group	33.83 ± 9.57	2.54 ± 0.33	33.28 ± 8.85	2.67 ± 0.37	34.30 ± 8.85	2.61 ± 0.33
Cohen’s d	-0.11 (6.30; 4.73)	0.67 (0.44; 0.83)	-0.10 (-4.20; 4.38)	0.10 (-0.10; 0.29)	-0.05 (-6.65; 4.43)	-0.03 (-0.17; 0.13)

PSV: peak systolic velocity.

Subsequent analysis revealed significant differences in blood velocity between the three pregnancy stages. According to Cohen^14^ and Wolf,^15^ effect size is interpreted as follows: > 0.20 small, > 0.25 educationally significant, > 0.50 moderate, practically and clinically significant, > 0.80 large.^5^ Changes to peak systolic velocity in the popliteal vein were found to be small in the experimental group (Table 4), whereas they were found to be clinically significant in the control group. The differences in cross-sectional area of the popliteal vein between particular data collection sessions were not found to be significant in any of the groups (Table 5).

**Table 4:** Differences in peak systolic velocity (cm/s) between particular data collection sessions with Cohen’s d values and their confidence intervals.

**PSV**	**Sessions 1 to 2**		**Sessions 2 to 3**		**Sessions 1 to 3**	
	**Cohen’s d**	**CI**	**Cohen’s d**	**CI**	**Cohen’s d**	**CI**
Experimental group	-0.38	(-0.54; -0.19)	0.17	(-0.01; 0.34)	-0.21	(-0.38; -0.05)
Control group	0.21	(-0.02; 0.42)	0.32	(0.11; 0.46)	0.55	(0.32; 0.69)

PSV: peak systolic velocity; CI: confidence interval.

**Table 5:** Differences in cross-sectional area (cm^2^) between particular data collection sessions with Cohen’s d values and their confidence intervals.

**Area**	**Sessions 1 to 2**		**Sessions 2 to 3**		**Sessions 1 to 3**	
	**Cohen’s d**	**CI**	**Cohen’s d**	**CI**	**Cohen’s d**	**CI**
Experimental group	0.06	(-4.78; 4.54)	-0.12	(-4.59; 4.36)	-0.05	(-4.89; 4.43)
Control group	0.02	(-6.17; 4.12)	-0.12	(-4.23; 6.47)	-0.09	(-6.28; 6.50)

CI: confidence interval.

## DISCUSSION

Pregnancy affects lower extremity venous hemodynamics. An earlier study observed a significant increase in clinical symptoms and signs of venous insufficiency during uncomplicated pregnancies.^3^ Changes to veins that occur during pregnancy are often reversible and return to their pre-pregnancy values during the puerperium period, however, in some cases, they may cause permanent damage to the venous system.^16^ Notwithstanding, no consensus has been reached on the major factor in the pathophysiology of venous insufficiency.^17^ Therefore, the purpose of this study was to evaluate the possible beneficial effect of special footwear on venous blood flow changes in pregnancy, as measured by Doppler.

In our study, no significant change in the cross-sectional area of the popliteal vein between 25, 30, and 35 weeks of gestation was found in either the control or the experimental group. This finding is consistent with a previous study by Ropacka-Lesiak et al.,^16^ who also found that the transverse diameter of the popliteal vein remained unchanged between different phases of pregnancy. A different study, focusing on pregnant patients with preexisting unilateral deep venous obstruction, did observe changes in popliteal vein diameter, but those changes were found to be inconsistent.^18^

A clinically significant reduction in the peak systolic velocity of the popliteal vein was observed from week 25 to week 35 of pregnancy in the control group. This finding is consistent with a previous study by Ropacka-Lesiak et al.,^1^ who also observed a gradual reduction of blood flow in the popliteal vein with advancing phases of pregnancy. In contrast to the decreased PSV found in the control group, significant increases in peak systolic velocity were observed in women in the experimental group who were provided with the special footwear, especially between the first and second measurements. A similar effect on the venous blood flow was observed in pregnant women wearing balanced inclined shoes in a previous study by Jang et al.^10^ In a study by Sousa et al.,^19^ who was testing unstable shoes on non-pregnant subjects, an increase in popliteal vein blood flow velocity was found in participants wearing the footwear tested. The increase in venous return in participants wearing the unstable shoes was associated with the increased gastrocnemius muscle activity in their study. The venous hemodynamics of the lower limb may be improved by muscle pumps, comprising the foot, calf and thigh muscle pump. The calf muscle pump has been found to be the most efficient. Moreover, calf muscle pump strength and ankle mobility training have been reported to improve venous hemodynamics in patients with chronic venous insufficiency.^19^^,^ 20 A complementary mechanism to the calf muscle pump is a plantar venous muscle pump that plays an important role in venous return by moving the blood in the foot venous reservoir upwards. The plantar muscle pump is activated with each step during walking, especially during the heel contact and stance phases of the gait cycle, when venous return is stimulated by a weight bearing compression of the plantar veins and muscular contraction around the veins.^21^^,^ 22 The footwear tested in our study is assumed to improve the blood circulation by strengthening the lower limb foot and/or calf muscle pumps, since the design of the insole tested stimulates activation of the foot muscles. Nevertheless, there is a need for further investigation of the beneficial effect on venous flow of the footwear tested and its applications, since there are many factors affecting the pregnant body, demonstrated by a significant difference between PSV in the experimental and control groups at the first data collection session, before the test footwear had been worn.

## CONCLUSION

The findings of this study have confirmed the occurrence of changes in venous flow during uncomplicated pregnancies. With advancing phases of pregnancy, the peak systolic velocity in the popliteal vein decreased significantly in the control group, potentially increasing the risk of venous disease development. However, it was demonstrated in the experimental group that wearing the J Hanák R, Ltd. footwear that was tested may prevent venous blood velocity reduction during advanced phases of pregnancy. Notwithstanding, there is a need for further investigation of the possible application of the footwear tested and its beneficial effect on venous flow in both pregnant and non-pregnant patients with venous disease
